# Teleophthalmology case study: Sankara Nethralaya, India

**Published:** 2022-06-07

**Authors:** Rachapalle Reddi Sudhir

**Affiliations:** 1 Head: Department of Preventive Ophthalmology and Medical Informatics, Sankara Nethralaya, Medical and Vision Research Foundation, Chennai, India.

**Sankara Nethralaya eye hospital offers free community-based teleophthalmology services as well as paid online services to existing and new patients – even those who do not have their own access to the internet.**

In 2003, Sankara Nethralaya eye hospital in Chennai became the first eye hospital in India to start using teleophthalmology to provide primary eye care to people in rural villages. This free service involves using a satellite link mounted on the roof of a mobile eye care van and includes comprehensive eye examinations and screening for cataract and diabetic retinopathy.

## New teleophthalmology models

With the arrival of the COVID-19 pandemic in 2020, it soon became clear that more people would need teleophthalmology services – not just those in rural areas.

The practice guidelines for telemedicine provided by the government of India at the beginning of the COVID-19 pandemic^1^ provided a framework for the regulation and diversification of teleconsultation services in the country. In response, and in addition to its existing service in rural areas, Sankara Nethralaya set up three new teleophthalmology access points: for new patients, existing patients, and patients who are without internet access but can visit an optical shop.

**Figure 1:**
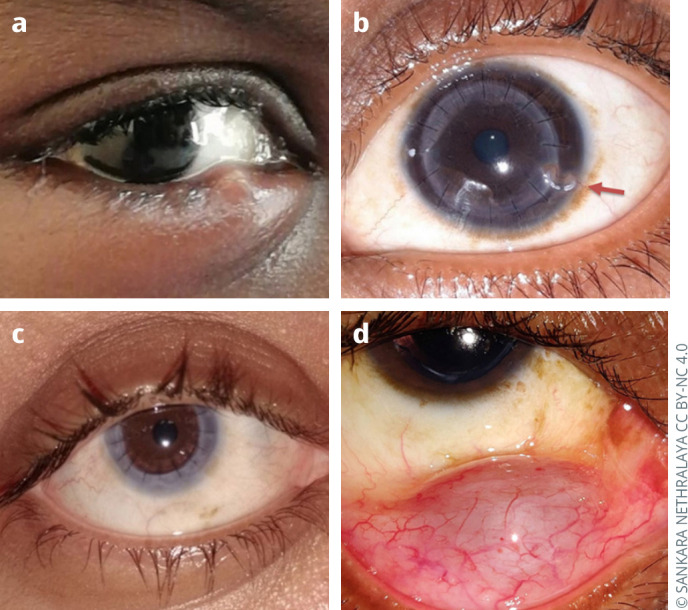
Photographs patients have taken of their own eyes after watching a video on how to take good quality photographs. **a** Hordeolum externum (stye). **b** Loose sutures following corneal graft. **c** Follow-up photo of deep anterior lamellar keratoplasty (DALK). **d** Conjunctival cyst. **INDIA**

**Figure:**
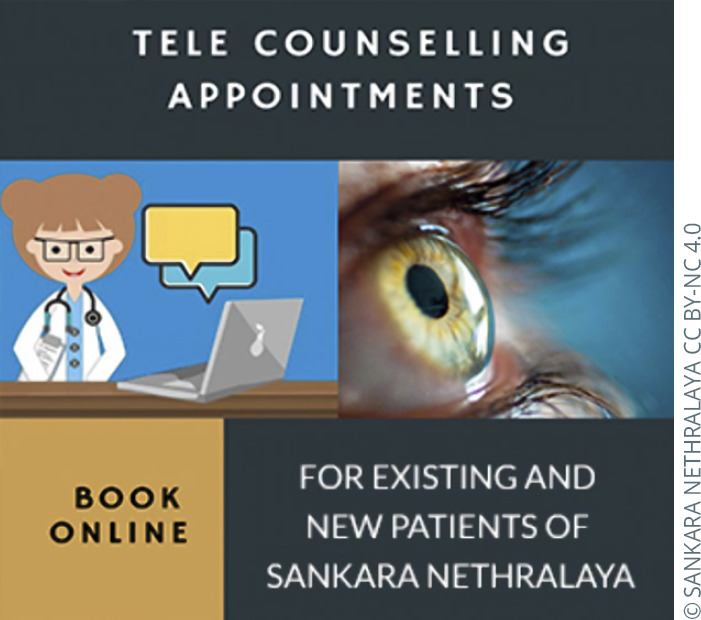
Patients who have internet access can book online video appointments. **INDIA**

1. **Existing patients.** These patients can set up an online teleconsultation appointment from home, by visiting the Senkara Nethralaya hospital website and completing an online form that is automatically linked to their patient records. Patients fill in their eye health history, check their vision using either of the two smartphone-based applications we recommend (Peek Acuity^2^ or EyeChart), take photos of the affected eye on their smartphone, and upload the results through the online patient portal link provided. Patients are encouraged to watch a video that shows them how to take good quality photographs (see Figure 1).
2. **New patients.** The process is like the one for existing patients, but an optometrist connects with the patient online to help them with documentation, uploading of old reports, taking pictures, and the triage process.
3. **Patients with no internet access, but who can visit an optical shop that is connected to the base hospital,** can make use of the shop's comprehensive eye examination facilities and connect to an ophthalmologist via audio/video call. This is currently a pilot project involving selected optical shops across India.

## Benefits of teleconsultation

The model of connecting a patient with an ophthalmologist directly from home requires relatively few resources. The hospital needs to have an electronic medical records system, teleconsultation facilities (audio/video calling) and a payment gateway. Free teleophthalmology services can be extended to those who cannot afford to pay after their eligibility is checked at the hospital.

With this model, patients need to have a smartphone and an internet connection (mobile internet or otherwise). Patients without access to a smartphone cannot share medical reports or pictures, but they can still get medical advice via short message service (SMS) and audio calls, or they can visit an optical shop linked to the hospital, if there is one nearby.

Teleconsultations are helpful for following up on surgical patients, for second opinions, for reviewing uploaded patient reports, and for counselling prior to surgery. Teleconsultations can also be used for orthoptic, contact lens, low vision, rehabilitation, and genetic counselling services. Collecting feedback from patients after every teleconsultation and taking quick action on grievances will help the quality management team to improve services.
